# Systematic Review: Adjuvant Chemotherapy for Locally Advanced Rectal Cancer with respect to Stage of Disease

**DOI:** 10.1155/2015/710569

**Published:** 2015-02-08

**Authors:** Shahab Hajibandeh, Shahin Hajibandeh

**Affiliations:** ^1^General Surgery Department, Pilgrim Hospital, Sibsey Road, Boston, Lincolnshire PE21 9QS, UK; ^2^General Surgery Department, Blackpool Victoria Hospital, 38 Whinney Heys Road, Blackpool, Lancashire FY3 8NR, UK

## Abstract

*Background*. Recent meta-analysis of 21 randomised controlled trials (RCTs) supports the use of adjuvant chemotherapy for nonmetastatic rectal carcinoma. In order to define a subgroup of patients who can potentially benefit from postoperative adjuvant chemotherapy, this study aims to review trials investigating adjuvant chemotherapy with respect to stage of disease in patients with locally advanced rectal cancer who had undergone surgery for cure (stage II and stage III). *Methods*. We searched electronic information sources to identify randomised trials evaluating adjuvant chemotherapy in patients with stages II and III rectal cancer with overall survival or disease-free survival as outcomes. Scottish Intercollegiate Guidelines Network notes on methodology were used to assess the methodological quality of the selected studies. Random-effects models were applied to calculate pooled outcome data. *Results*. Eight studies reporting total of 5527 patients were selected for analysis. Adjuvant chemotherapy was associated with statistically significant improvement in disease-free survival and overall survival compared to surgery alone in both stage II and stage III cancer. *Conclusions*. This study indicates that both stage II and stage III rectal cancer patients may benefit from postoperative adjuvant chemotherapy. However, the benefits of adjuvant chemotherapy for patients who already had neoadjuvant chemoradiation still remain unknown.

## 1. Introduction

Colorectal cancer is a major cause of morbidity and mortality throughout the world [1]. It accounts for over 9% of all cancer incidences. It is the third most common cancer worldwide and the fourth most common cause of death [2, 3]. Rectal cancer is defined as disease occurring in the distal 12–15 cm of the large bowel, where the distal two-thirds is extraperitoneal [4]. Surgery is the mainstay treatment of resectable rectal cancer. Total mesorectal excision (TME) is the standard surgical approach to proctectomy for rectal cancer and is associated with reduced local recurrence rate and improved oncologic outcomes [5].

In locally advanced stages of rectal carcinoma, stage II (T3-4, N0, M0) and stage III (any T, N1-2, M0), surgery is often supported by combined modality therapy to further reduce the risk of local and distant recurrence [6]. It has been shown that preoperative chemoradiotherapy is associated with reduced local recurrence rate and it is considered as the standard of care for moderate or high-risk resectable rectal cancer [7, 8].

Although adjuvant chemotherapy is recommended for stage III and high-risk stage II colon cancer, uncertainty remains around the benefits of such chemotherapy for patients with stage II and III rectal cancer [7]. The most recent meta-analysis of 21 randomised controlled trials (RCTs), conducted by Cochrane Colorectal Cancer Group, supports the use of 5-fluorouracil (5-FU) based postoperative adjuvant chemotherapy for patients undergoing apparently radical surgery for nonmetastatic rectal carcinoma. In fact, it was reported that adjuvant chemotherapy is associated with reduction in the risk of death and risk of disease recurrence in rectal cancer [6]. However, this study did not provide adequate evidence about outcomes of adjuvant chemotherapy with respect to stage of rectal cancer.

Whether all patients with locally advanced rectal cancer should receive adjuvant chemotherapy is still controversial. Knowledge about outcomes of adjuvant chemotherapy with respect to stage of rectal cancer is required in order to be able to define a subgroup of patients who can potentially benefit from postoperative adjuvant chemotherapy. Therefore, this study aims to review trials investigating adjuvant chemotherapy with respect to stage of disease in patients with locally advanced rectal cancer who had undergone surgery for cure (stage II and stage III).

## 2. Methods

### 2.1. Search Strategy

In order to find appropriate articles about adjuvant chemotherapy in rectal cancer, Ovid Medline (1946 to February 2014), PubMed, and the Cochrane library were used as online databases.

In Medline, The keyword “adjuvant chemotherapy” and medical subject headings (MeSH) term “chemotherapy, adjuvant” were combined by OR (search A). Also, keyword “rectal cancer” and MeSH term “rectal neoplasms” were combined by OR (search B). The resulted literatures from search A and search B were combined by AND in order to narrow the results. Then, the resulted search was limited to randomized controlled trial.

In PubMed, search strategy consisted of “adjuvant chemotherapy” and “rectal cancer.” Then, the resulted search was limited to randomized controlled trial.

In order to reduce the possibility of missing relevant articles, the reference lists of relevant articles were reviewed.

### 2.2. Study Selection

The title, abstract, and introduction sections of the obtained literatures were assessed carefully by two independent reviewers (Shahab Hajibandeh and Shahin Hajibandeh) to find relevant articles. Full texts of relevant reports were retrieved and those articles that met the inclusion criteria of the study were selected. Any discrepancies in inclusion were resolved by discussion between the reviewers. If necessary, an independent third reviewer was consulted.

### 2.3. Inclusion Criteria

Inclusion criteria are as follows:

- randomised controlled trials,
- including patients with stage II or/and stage III resectable rectal cancer as population of interest,
- comparing adjuvant chemotherapy with curative surgery alone as interventions of interest,
- investigating overall survival (OS) or/and disease-free survival (DSF) as outcome measures,
- reporting survival outcomes stratified to stage II or/and to stage III rectal cancer.

### 2.4. Exclusion Criteria

Exclusion criteria are as follows:

- previous neoadjuvant cancer therapy (radiotherapy or chemotherapy),
- review articles,
- nonrandomised observational studies,
- case reports,
- case series,
- clinical audits,
- ongoing trials,
- authors' replies,
- language other than English.

### 2.5. Data Extraction

The data from the included articles were extracted on data extraction sheets by two independent reviewers (Shahab Hajibandeh and Shahin Hajibandeh). The extracted data included publication year, sample size, study design, patient characteristics, type of patients, type of intervention, and outcomes. Disagreements were resolved by discussion between the two reviewers. If no agreement could be reached, a third reviewer made the final decision.

### 2.6. Methodological Quality

The methodological quality of the included articles was assessed by two independent reviewers (Shahab Hajibandeh and Shahin Hajibandeh) using SIGN (Scottish Intercollegiate Guidelines Network) notes on methodology [9, 10] which consists of two sections and classifies each study as high quality, acceptable, or low quality. Disagreements were resolved by discussion between the two reviewers. If no agreement could be reached, a third reviewer made the final decision.

### 2.7. Statistical Analysis and Data Synthesis

The outcomes in our review (DFS and OS) were dichotomous variables; therefore, the odds ratio (OR), which is the odds of survival in the chemotherapy group compared to surgery only group, was calculated as the summary measure. An OR of less than one would favour the adjuvant chemotherapy. Separate analyses were performed for stage II and stage III rectal cancer. The unit of analysis in our review was the individual patient.

We assessed heterogeneity among the studies using Cochrane chi-squared (*χ* ^2^, or Chi^2^) test and quantified inconsistency by calculating *I* ^2^. The Review Manager 5.3 was used for data synthesis. We used random effect modelling for analysis and reported the results in a forest plot with 95% confidence intervals (CI).

## 3. Results

**Figure 1:**
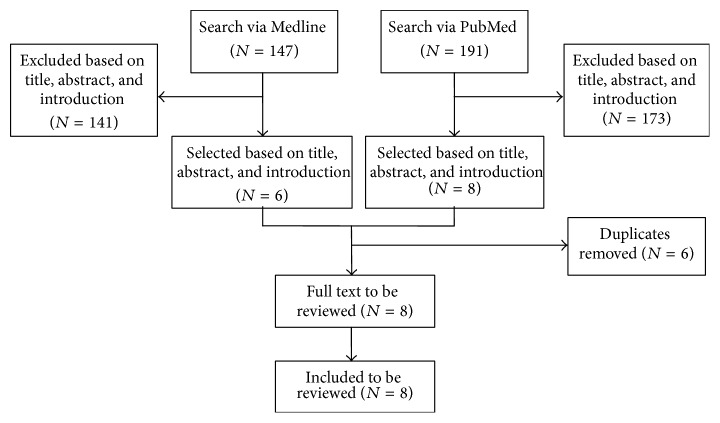
Flowchart for the review.

Searches of electronic information sources identified 147 and 191 articles in Medline and PubMed, respectively, of which 8 studies (Glimelius et al. 2005 [11], CCCSGJ 1995 [12], Fisher et al. 1988 [13], Kato et al. 2002 [14], Kodaira et al. 1998 [15], Hamaguchi et al. 2011 [16], QUASAR 2007 [17], and Sakamoto et al. 2007 [18]) were found to be eligible for this review (Figure 1 and Table 1).

### 3.1. Included Population

The included studies enrolled a total number of 11839 colorectal cancer patients of which 5527 patients had stage II or stage III rectal cancer. Forty seven percent of rectal cancer patients had stage II disease and 53% of them had stage III disease. A sum of 2954 (53%) rectal cancer patients received adjuvant chemotherapy and 2573 (47%) patients were treated with surgery alone. None of the included patients had received neoadjuvant chemotherapy or radiotherapy (Table 2).

### 3.2. Included Studies

Glimelius et al. 2005 [11] is a 4-arm multicentre RCT which included 2224 patients with stage II or stage III resectable colorectal cancer. The exclusion criteria of this study were another malignancy (except squamous cell carcinoma of the skin and cervical carcinoma stage 0), previous chemotherapy or radiotherapy, severe cardiopulmonary disease, and major laboratory abnormalities. There were 3 intervention arms (A, B, and C) in this study that received postoperative adjuvant chemotherapy. Arm A received 5FU plus levamisole, arm B received 5FU plus leucovorin, and arm C received 5FU plus leucovorin plus levamisole. Patients in control arm were treated with curative surgery only. OS was outcome measure of this study that was analysed by log-rank test.

CCCSGJ 1995 [12] is a 6-arm multicentre RCT which included 2001 patients (997 colon cancer and 1004 rectal cancer) with stage II or stage III resectable colorectal cancer. The exclusion criteria of this study were age over 75, serious complications, history of cancer therapy (surgery, radiotherapy, chemotherapy, etc.), synchronous or metachronous multiple primary carcinomas, and major laboratory abnormalities. There were 3 rectal cancer arms in this study. One of the intervention arms received intraoperative intra-arterial mitomycin C (MMC) and postoperative adjuvant MMC plus 5FU. The other intervention arm received adjuvant MMC plus 5FU. Patients in control arm were treated with curative surgery only. OS and DFS were outcome measures of this study that were analysed by Kaplan-Meier method, log-rank test, and Cox regression model.

Fisher et al. 1988 [13] is a 3-arm multicentre RCT which included 555 patients with stage II or stage III resectable rectal cancer. The exclusion criteria of this study were stage I and stage IV rectal cancer, previous cancer, second primary cancer in the colon and abnormal performance status, and hematologic profile. There were 2 intervention arms in this study. One arm received adjuvant chemotherapy with 5-FU plus semustine plus vincristine and the other arm received adjuvant radiotherapy. Patients in control arm were treated with curative surgery only. OS and DFS were outcome measures of this study.

Kato et al. 2002 [14] is a 2-arm multicentre RCT which included 289 patients with stage II or stage III resectable colorectal cancer. The exclusion criteria of this study were age over 75, anticancer therapy (chemotherapy, radiation therapy, immunotherapy, or a combined modality of these) after the surgery, synchronous or metachronous double cancer, synchronous or metachronous multiple colorectal cancer (except for intramucosal carcinoma), abnormal performance status, and major laboratory abnormalities. Patients in intervention arm received adjuvant chemotherapy with Tegafur-uracil (UFT) and patients in control arm were treated with curative surgery only. OS and DFS were outcome measures of this study that were analysed by Kaplan-Meier method and log-rank test.

Kodaira et al. 1998 [15] is a 2-arm multicentre RCT which included 834 patients with stage II or stage III resectable rectal cancer. The exclusion criteria of this study were age over 70, serious complications, other surgical therapies, radiotherapy, chemotherapy or immunotherapy (alone or in combination), synchronous or metachronous multiple primary carcinomas, and major laboratory abnormalities. Patients in intervention arm received adjuvant chemotherapy with MMC plus UFT and patients in control arm were treated with curative surgery only. OS and DFS were outcome measures of this study that were analysed by Kaplan-Meier method and log-rank test.

Hamaguchi et al. 2011 [16] is a 2-arm multicentre RCT which included 276 patients with stage III resectable rectal cancer. The exclusion criteria of this study were age under 20 or above 75, abnormal performance status, and major laboratory abnormalities. Patients in intervention arm received adjuvant chemotherapy with UFT and patients in control arm were treated with curative surgery only. OS and DFS were outcome measures of this study that were analysed by Kaplan-Meier method, log-rank test, and Cox proportional hazards models.

QUASAR 2007 [17] is a 2-arm multicentre RCT which included 3239 patients with stage II or stage III resectable colorectal cancer. The exclusion criteria of this study were distant metastases, definite contraindications to chemotherapy, and prior chemotherapy. Patients in intervention arm received adjuvant chemotherapy with 5-FU plus L-folinic acid and patients in control arm were treated with curative surgery only. OS and DFS were outcome measures of this study that were analysed by log-rank methods.

Sakamoto et al. 2007 [18] is an individual patient meta-analysis that included 2091 patients with resectable rectal cancer from 5 RCTs. In this study, patients in intervention group had received adjuvant chemotherapy with UFT and patients in control group had been treated by curative surgery only. OS and DFS were outcome measures of this study. The main reason for including Sakamoto 2007 was the fact that it provided survival data stratified to rectal cancer stages from 2 RCTs (JFMC15-1, JFMC15-2 [19]) that their original reports did not provide any data stratified to stages of rectal cancer.

### 3.3. Outcomes

#### 3.3.1. Disease-Free Survival

DFS is defined as time from randomization until recurrence, death without recurrence, or occurrence of a new primary cancer. All the included studies, except Glimelius et al. 2005 [11], reported DFS as outcome measure (Table 3). DFS stratified according to stages II and III rectal cancer has been reported by CCCSGJ 1995 [12], Fisher et al. 1988 [13], Kato et al. 2002 [14], Kodaira et al. 1998 [15], and Sakamoto et al. 2007 [18]. DSF reported by Hamaguchi et al. 2011 [16] was related to stage III only and QUASAR 2007 [17] reported DSF stratified to stage II only.

*Stage II Disease.* In CCCSGJ 1995 [12], there was statistically significant difference in DFS between chemotherapy groups and surgery only group for stage II rectal cancer (arm 1 versus surgery: 85.4% versus 62.7%, *P* = significant; arm 2 versus surgery: 78.8% versus 62.7%, *P* = significant). Fisher et al. 1988 [13] also showed significantly better DFS in chemotherapy group compared to control group (61% versus 39%, *P* = significant). This was consistent with results from Kato et al. 2002 [14] (87.8% versus 50%, *P* = significant), QUASAR 2007 [17] (82.9% versus 76.7%, *P* = significant), and Sakamoto et al. 2007 [18] (77.1% versus 66.4%, *P* = significant). However, unlike the above studies, Kodaira et al. 1998 [15] did not report statistically significant difference in DFS between chemotherapy and control groups for stage II disease (77.3% versus 67.5%, *P* = not significant). 

*Stage III Disease.* In CCCSGJ 1995 [12], adjuvant chemotherapy resulted in significantly better DFS only in one of the intervention arms (arm 1 versus surgery: 53.1% versus 39.3%, *P* = not significant; arm 2 versus surgery: 62.9% versus 39.3% *P* = significant). The better DFS in chemotherapy group compared to surgery only group was also reported by Fisher et al. 1988 [13] (29% versus 25%, *P* = significant), Kato et al. 2002 [14] (65% versus 37.1%, *P* = significant), Kodaira et al. 1998 [15] (54.5% versus 40.7%, *P* = significant), Hamaguchi et al. 2011 [16] (68.9% versus 56.3%, *P* = significant), and Sakamoto et al. 2007 [18] (55% versus 46.5%, *P* = significant).

#### 3.3.2. Overall Survival

OS is defined as time from randomization until death from any cause. All the included studies reported OS as outcome measure (Table 4). Glimelius et al. 2005 [11], CCCSGJ 1995 [12], Fisher et al. 1988 [13], Kodaira et al. 1998 [15], and Sakamoto et al. 2007 [18] reported OS stratified according to stages II and III disease. OS reported by Hamaguchi et al. 2011 [16] was related to stage III only. QUASAR 2007 [17] reported OS related to stage II only. Kato et al. 2002 [14] reported OS stratified to all rectal cancers but not stratified to specific stage.

*Stage II Disease.* Adjuvant chemotherapy resulted in better OS compared to surgery only for stage II disease in Fisher et al. 1988 [13] (80% versus 57%, *P* = significant), QUASAR 2007 [17] (80.9% versus 76.7%, *P* = significant), Sakamoto et al. 2007 [18] (82.4% versus 76.8%, *P* = significant), and one of the intervention arms in CCCSGJ 1995 [12] (arm 1 versus surgery: 82.2% versus 68.1%, *P* = significant; arm 2 versus surgery: 81.1% versus 68.1% *P* = not significant). However, there was no statistically significant difference in OS between two groups in Glimelius et al. 2005 [11] (81% versus 73%, *P* = not significant) and Kodaira et al. 1998 [15] (80.4% versus 75.9%, *P* = not significant).

*Stage III Disease.* There was statistically significant difference in OS between chemotherapy and surgery only groups for stage III disease in Hamaguchi et al. 2011 [16] (85.3% versus 72.1%, *P* = significant), Sakamoto et al. 2007 [18] (64.1% versus 59.2%, *P* = significant), and one of the intervention arms in CCCSGJ 1995 [12] (arm 1 versus surgery: 54.7% versus 43.1%, *P* = not significant; arm 2 versus surgery: 62.3% versus 43.1% *P* = significant). Unlike the above studies, there was no statistically significant difference in OS between two groups in Fisher et al. 1988 [13] (37% versus 35%, *P* = not significant), Glimelius et al. 2005 [11] (48% versus 51%, *P* = not significant), and Kodaira et al. 1998 [15] (53.4% versus 49.1%, *P* = not significant).

### 3.4. Methodological Quality and Risk of Bias

Based on SIGN notes on methodology checklist, the included studies had high methodological quality. In all the included RCTs, an appropriate and clearly focused question was addressed, the assignment of subjects to treatment groups was randomised, an adequate concealment method was used, the treatment and control groups were similar at the start of the trial, the only difference between groups was the treatment under investigation, and all relevant outcomes were measured in a standard, valid, and reliable way.

In terms of risk of bias, Glimelius et al. 2005 [11], CCCSGJ 1995 [12], Fisher et al. 1988 [13], Kato et al. 2002 [14], Kodaira et al. 1998 [15], Hamaguchi et al. 2011 [16], and QUASAR 2007 [17] were associated with low risk of reporting and selection bias. Because of nature of study, Sakamoto et al. 2007 [18] were associated with high risk of reporting bias but low risk of any other bias.

### 3.5. Odds Ratio Analysis and Outcome Synthesis

#### 3.5.1. Stage II Disease

*Disease-Free Survival.* DFS was reported in 2366 patients. Odds ratio analysis showed that patients receiving adjuvant chemotherapy had better DFS than patients treated by surgery alone [OR = 0.51 (95% CI 0.39–0.67), *P* < 0.00001]. Moderate heterogeneity among the studies existed (*I* ^2^ = 44%, *P* = 0.11).

*Overall Survival.* OS was reported in 2637 patients. Odds ratio analysis showed that patients receiving adjuvant chemotherapy had better DFS than patients treated by surgery alone [OR = 0.64, (95% CI 0.51–0.80), *P* < 0.0001]. Low heterogeneity among the studies existed (*I* ^2^ = 24%, *P* = 0.25).

#### 3.5.2. Stage III Disease

*Disease-Free Survival.* DFS was reported in 2470 patients. Odds ratio analysis showed that patients receiving adjuvant chemotherapy had better DFS than patients treated by surgery alone [OR = 0.61 (0.51–0.73), *P* < 0.00001]. Low heterogeneity among the studies existed (*I* ^2^ = 12%, *P* = 0.34).

*Overall Survival.* OS was reported in 2761 patients. Odds ratio analysis showed that patients receiving adjuvant chemotherapy had better DFS than patients treated by surgery alone [OR = 0.76 (0.61–0.96), *P* < 0.02]. Moderate heterogeneity among the studies existed (*I* ^2^ = 47%, *P* = 0.09).

**Figure 2:**
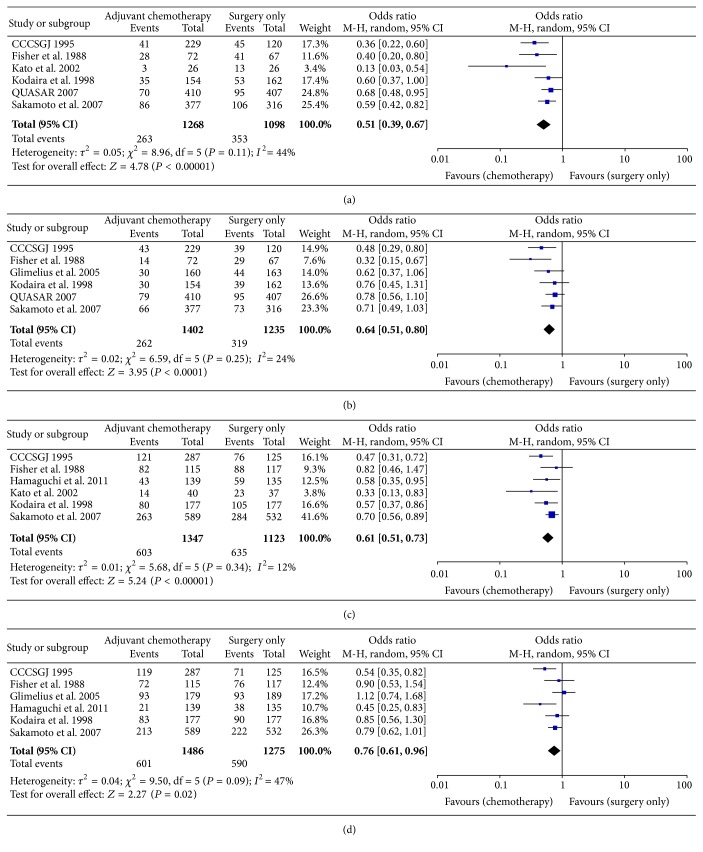
Forest plots of comparison of (a) stage II DFS, (b) stage II OS, (c) stage III DFS, and (d) stage III OS. The solid squares denote the odds ratios (ORs), the horizontal lines represent the 95% confidence intervals (CIs), and the diamond denotes the pooled OR. M-H, Mantel Haenszel test.

Results of synthesis of the outcome parameters are depicted in Figure 2.

## 4. Discussion

Adjuvant chemotherapy is standard of care for stage III and high-risk stage II colon cancer [7]. It has been shown that 5-FU based adjuvant chemotherapy can be beneficial in locally advanced rectal cancer as well [6]. However, which group of patients with locally advanced rectal cancer can benefit from adjuvant chemotherapy still remains a question.

In this review, our pooled analysis of data from seven RCTs and one individual patient meta-analysis, enrolling total number of 5527 patients, found that adjuvant chemotherapy is associated with better DFS and OS in both stage II and stage III rectal cancers. There was low to moderate heterogeneity among the studies in our analysis that can be partly explained by different chemotherapy regimens and some differences in baseline characteristics of the included studies. There was not considerable inconsistency in the direction of effect by adjuvant chemotherapy among the included studies. We used a random-effects meta-analysis to incorporate heterogeneity that cannot be explained although this is not a substitute for a thorough investigation of heterogeneity. Nevertheless, we do not believe that our results have been affected by between-study heterogeneity significantly.

Our analysis showed that for stage III rectal cancer improvement in DFS was more considerable than improvement in OS. This may be explained by the fact that marginally significant DFS advantages may not translate into OS benefit [20]. Considering that DFS is more appropriate end point than OS in stage III disease, demonstration of a clinically meaningful improvement in DFS may be adequate evidence of clinical benefit [21]. Moreover, improved survival after adjuvant chemotherapy in patients with stage III rectal cancer has been reported by prospective cohort studies [22, 23].

The included population in our analysis did not receive preoperative treatment with chemoradiation which has recently become the standard of care in patients with stages II and III rectal cancer in Europe and in the USA. However, although preoperative chemoradiotherapy inhibits local recurrence and reduces toxicity, it does not improve OS compared with postoperative chemoradiotherapy [24]. This highlights the importance of knowledge about effect of adjuvant chemotherapy on survival in rectal cancer patients despite common practice in western countries. Postoperative adjuvant chemotherapy for stages II and III rectal cancer has been recommended by National Institutes of Health (NIH) consensus conference [25] and our findings support this recommendation.

Despite a comprehensive literature search, we identified only 8 studies that provided data separately for stages II and III rectal cancer. However, considering the proven benefits of neoadjuvant chemoradiotherapy in preventing local recurrence of disease, it is unlikely to identify further studies with rectal cancer patients without preoperative chemoradiotherapy at least in western countries.

In our review, the included studies used conventional chemotherapy agents such as UFT and 5FU and none of them used modern chemotherapy agents such as oxaliplatin, irinotecan, or bevacizumab which can significantly improve the therapeutic efficacy of conventional chemotherapy [26] and improve survival [27]. Therefore, the effect of adjuvant chemotherapy on survival outcomes may be greater with modern agents.

Our review has some limitations. The included population in our review did not receive preoperative chemoradiotherapy which is currently the standard treatment for rectal cancer; therefore, the benefits of adjuvant chemotherapy for patients who already had neoadjuvant chemoradiation still remain unknown. The chemotherapy regimens used in the included studies were heterogeneous although all of them were 5FU based. Moreover, due to unavailability of original stratified data from two RCTs, one individual patient data meta-analysis, which contained relevant data, was included. Although it was a high quality study, it was inevitably associated with reporting bias. Some of the included studies were not specifically designed for stage II or stage III rectal cancers and they included patients with colon cancer as well; therefore, their stratified data were used for analysis. All of these may affect robustness of the results of our review and can subject it to bias.

## 5. Conclusions and Future Directions

Our study indicates that both stage II and stage III rectal cancer patients may benefit from postoperative adjuvant chemotherapy. It is associated with statistically significant improvement in disease-free survival and overall survival compared to surgery alone in both stage II and stage III cancer. There was no significant heterogeneity between the included studies in terms of eligibility criteria, outcomes, and design. This can potentially make the conclusion of our study reliable.

Considering heterogeneity between included studies in terms of chemotherapy agents and regimens, further RCTs are required to compare different chemotherapy agents and regimens in stage II and stage III rectal cancer. The future randomised trials should focus on effect of modern chemotherapy agents as adjuvant therapy in stage II and stage III rectal cancer patients who have already received neoadjuvant treatments. Moreover, future trials should include patients specifically with stage II and stage III rectal cancer to provide further evidence about benefits of adjuvant chemotherapy in these subgroups.

## Figures and Tables

**Table 1:** Main characteristics of included studies.

	Glimelius et al. 2005 [11]	CCCSGJ 1995 [12]	Fisher et al. 1988 [13]	Kato et al. 2002 [14]	Kodaira et al. 1998 [15]	Hamaguchi et al. 2011 [16]	QUASAR 2007 [17]	Sakamoto et al. 2007 [18]
Study design	Multicentre RCT	Multicentre RCT	Multicentre RCT	Multicentre RCT	Multicentre RCT	Multicentre RCT	Multicentre RCT	Patient based meta-analysis of multicentre RCTs

Number of patients	2224	2001	555	289	834	606	3239	2091

Stage of rectal cancer	II and III	II and III	II and III	II and III	II and III	III	II and III	II and III

Intervention arm(s)	Surgery adjuvant CT	Arm 1: surgery adjuvant intra-arterial + systemic CT arm 2: surgery adjuvant CT	Arm 1: surgery adjuvant CT arm 2: surgery adjuvant RD	Surgery adjuvant CT	Surgery adjuvant CT	Surgery adjuvant CT	Surgery adjuvant CT	Surgery adjuvant CT

Control arm	Surgery	Surgery	Surgery	Surgery	Surgery	Surgery	Surgery	Surgery

Chemotherapy regimen	Arm 1: 5FU + levamisole arm 2: 5FU + leucovorin arm 3: 5FU + leucovorin + levamisole	Arm 1: MMC + 5FU arm 2: MMC + 5FU	5-FU + semustine + vincristine	UFT	MMC + UFT	UFT	5-FU + L-folinic acid	UFT

Intention to treat analysis	Yes	Not reported	Not reported	Not reported	Not reported	Not reported	Yes	Not reported

Outcomes	OS	DSF and OS	DSF and OS	DSF and OS	DSF and OS	DSF and OS	DSF and OS	DSF and OS

Risk of bias	Low	Low	Low	Low	Low	Low	Low	Low (except reporting bias)

Methodological quality^*^	High	High	High	High	High	High	High	High

CT: chemotherapy, RCT: randomised controlled trial, RD: radiotherapy, 5-FU: 5-fluorouracil, MMC: mitomycin C, UFT: Tegafur-uracil, OS: overall survival, and DSF: disease-free survival, ^*^based on SIGN notes on methodology checklist.

**Table 2:** Baseline characteristics of rectal cancer patients in included studies.

	Number of patients			Age	Male * *(%)	Stage II rectal cancer		Stage III rectal cancer		Previous treatment	
	Total	Chemotherapy (%)	Control (%)			Chemotherapy (%)	Control (%)	Chemotherapy (%)	Control (%)	Chemotherapy	Radiotherapy
Glimelius et al. 2005 [11]	691	339 (49)	352 (51)	64 versus 66	NR	160 (23)	163 (24)	179 (26)	189 (27)	No	No

CCCSGJ 1995 [12]	761	516 (68)	245 (32)	57 versus 60	NR	229 (30)	120 (16)	287 (38)	125 (16)	No	No

Fisher et al. 1988 [13]	371	187 (50)	184 (50)	61 versus 61	240 (64)	72 (19)	67 (18)	115 (31)	117 (32)	No	No

Kato et al. 2002 [14]	129	66 (51)	63 (49)	60.2 versus 61.4	NR	26 (20)	26 (20)	40 (31)	37 (29)	No	No

Kodaira et al. 1998 [15]	670	331 (49)	339 (51)	NR	NR	154 (23)	162 (25)	177 (26)	177 (26)	No	No

Hamaguchi et al. 2011 [16]	274	139 (51)	135 (49)	59 versus 58	165 (60)	NR	NR	139 (51)	135 (49)	No	No

QUASAR 2007 [17]	817	410 (50)	407 (50)	NR	NR	410 (50)	407 (50)	NR	NR	No	No

Sakamoto et al. 2007 [18]	1814	966 (53)	848 (47)	NR	NR	377 (21)	316 (17)	589 (33)	532 (29)	No	No

Total	5527	2954 (53)	2573 (47)	—	—	1428 (26)	1261 (23)	1526 (27)	1312 (24)	—	—

NR: not reported.

**Table 3:** Disease-free survival reported by included studies.

Study	DFS							
	Stage II				Stage III			
	Control	Intervention		Statistical significance	Control	Intervention		Statistical significance
CCCSGJ 1995 [12]	62.7%	Arm 1 85.4% *P* = S	Arm 2 78.8% *P* = S		39.3%	Arm 1 53.1% *P* = NS	Arm 2 62.9% *P* = S	

Fisher et al. 1988 [13]	39%	61%		S	25%	29%		S

Kato et al. 2002 [14]	50.0%	87.8%		S	37.1%	65.0%		S

Kodaira et al. 1998 [15]	67.5%	77.3%		NS	40.7%	54.5%		S

Hamaguchi et al. 2011 [16]	Not Reported	Not Reported			56.3%	68.9%		S

QUASAR 2007 [17]	76.7%	82.9%		S	Not Reported	Not Reported		

Sakamoto et al. 2007 [18]	66.4%	77.1%		S	46.5%	55.0%		S

DFS: disease-free survival, S: significant, and NS: not significant.

**Table 4:** Overall survival reported by included studies.

Study	OS							
	Stage II				Stage III			
	Control	Intervention		Statistical significance	Control	Intervention		Statistical significance
Glimelius et al. 2005 [11]	73%	81%		NS	51%	48%		NS

CCCSGJ 1995 [12]	68.1%	Arm 1 82.2% *P* = S	Arm 2 81.1% *P* = NS		43.1%	Arm 1 54.7% *P* = NS	Arm 2 62.3% *P* = S	

Fisher et al. 1988 [13]	57%	80%		S	35%	37%		NS

Kodaira et al. 1998 [15]	75.9%	80.4%		NS	49.1%	53.4%		NS

Hamaguchi et al. 2011 [16]	Not Reported	Not Reported			72.1%	85.3%		S

QUASAR 2007 [17]	76.7%	80.9%		S	Not Reported	Not Reported		

Sakamoto et al. 2007 [18]	76.8%	82.4%		S	59.2%	64.1%		S

OS: overall survival, S: significant, and NS: not significant.
